# Odor naming and interpretation performance in 881 schizophrenia subjects: association with clinical parameters

**DOI:** 10.1186/1471-244X-13-218

**Published:** 2013-08-30

**Authors:** Anne Kästner, Dörthe Malzahn, Martin Begemann, Constanze Hilmes, Heike Bickeböller, Hannelore Ehrenreich

**Affiliations:** 1 Clinical Neuroscience, Max Planck Institute of Experimental Medicine, Hermann-Rein-Str.3, 37075 Göttingen, GERMANY; 2 Department of Genetic Epidemiology of the University Medical Center, Göttingen, Germany; 3 DFG Research Center for Nanoscale Microscopy & Molecular Physiology of the Brain (CNMPB), Göttingen, Germany

**Keywords:** Odor naming, Higher olfactory processing, Odor interpretation, Positive symptoms, Cognition

## Abstract

**Background:**

Olfactory function tests are sensitive tools for assessing sensory-cognitive processing in schizophrenia. However, associations of central olfactory measures with clinical outcome parameters have not been simultaneously studied in large samples of schizophrenia patients.

**Methods:**

In the framework of the comprehensive phenotyping of the GRAS *(Göttingen Research Association for Schizophrenia)* cohort, we modified and extended existing odor naming (active memory retrieval) and interpretation (attribute assignment) tasks to evaluate them in 881 schizophrenia patients and 102 healthy controls matched for age, gender and smoking behavior. Associations with emotional processing, neuropsychological test performance and disease outcome were studied.

**Results:**

Schizophrenia patients underperformed controls in both olfactory tasks. Odor naming deficits were primarily associated with compromised cognition, interpretation deficits with positive symptom severity and general alertness. Contrasting schizophrenia extreme performers of odor interpretation (best versus worst percentile; N=88 each) and healthy individuals (N=102) underscores the obvious relationship between impaired odor interpretation and psychopathology, cognitive dysfunctioning, and emotional processing (all p<0.004).

**Conclusions:**

The strong association of performance in higher olfactory measures, odor naming and interpretation, with lead symptoms of schizophrenia and determinants of disease severity highlights their clinical and scientific significance. Based on the results obtained here in an exploratory fashion in a large patient sample, the development of an *easy-to-use* clinical test with improved psychometric properties may be encouraged.

## Background

The sense of smell and its relation to neurological and psychiatric diseases is a field of growing interest in clinical research. Like other neuropsychological measures, it provides the opportunity to assess brain function in a non-invasive way [1]. Central neural circuits underlying olfactory function can be mapped to temporolimbic and frontal brain regions [2]. Dysfunctional connectivity in these olfactory structures has been consistently implicated in the affective and cognitive symptomatology of schizophrenia patients [3]. Consequently, olfactory tasks serve as behavioral probes to assess the structural and functional integrity of neural substrates underlying disturbed sensory-cognitive and emotional processing in schizophrenia [3].

Olfactory input triggers cognitive events, often in form of autobiographical memories [4-6]. Tests measuring olfactory cognition address the disturbed integration of olfactory input into cognitive processing. They share the common principle of administering an odorant prior to the execution of related mental operations with varying degrees of difficulty [7]. The more demanding the olfactory task, the more likely the contribution of higher-order cortical olfactory circuits. Where in the olfactory projection cascade difficulties for schizophrenia individuals arise is still unclear [8]. Nevertheless, evidence of a peripheral deficit in terms of abnormal olfactory receptor function accumulated over the past years [9,10]. Profound problems consistently emerge in schizophrenia patients when the olfactory task requires basal cognitive operations like the passive recognition of a presented odor from lists of distractors (passive odor identification) [11-13], or when a delay between odor presentation and identification is introduced (odor memory) [4,14].

The identification and explicit naming of odorants (active odor naming) is a complex task requiring correct smell encoding, semantic memory, and selection of the most appropriate denomination [15]. Saoud and coworkers delivered first evidence of an odor naming deficit in a small sample (N=24) of male schizophrenia patients [16]. An implication of dorsolateral prefrontal executive functioning networks in this task was supported by its correlation with Wisconsin Card Sorting Test performance [16]. Surprisingly, clinical variables like PANSS scores or age at onset did not correlate with odor naming ability in this study [16]. Although less cognitively demanding, associations with schizophrenia psychopathology including cognition have been consistently shown for passive odor identification [8,17-19]. The missing link between PANSS scores and odor naming ability in the study by Saoud and coworkers likely reflects a lack of statistical power due to the very small sample size (N=24). As it is a very elegant and naturalistic measure of the integrity of prefrontal networks, we decided to extend in the present study the work reported by Saoud and colleagues by evaluating odor naming performance and its relationship with clinical and cognitive variables in a large sample of schizophrenia patients.

Olfactory stimuli do not only induce cognitive activity, they immediately evoke emotional reactions [20]. To explore the nature of affective states in response to different scents, available olfactory function tests require the judgement of various qualities of given odors. Schizophrenia subjects are less accurate when deciding whether an odor is edible, pleasant or familiar as compared to healthy controls [21-24]. It is known that the rating of gustatory or semantic properties of odors facilitates their subsequent naming in healthy control subjects [25]. No study has ever investigated whether schizophrenia patients have difficulties in judging gustatory (sweet, hot) or semantic properties (association of an odor with its context, i.e. technical or natural) of odors beyond familiarity, hedonicity and edibility ratings (odor interpretation). Moreover, it has not been evaluated how the inability to accurately interpret olfactory stimuli relates to schizophrenia symptomatology.

As a part of the very comprehensive phenotyping of the GRAS cohort [26], odor interpretation and naming was evaluated in 881 schizophrenia patients and 102 healthy matched controls. In the present study we assessed odor naming and interpretation ability using diverse odors typical for the ‘olfactory environment’ of German subjects as well as descriptive attributes (hedonic, gustatory and semantic). We evaluated the relationship of olfactory task performance with relevant symptom domains and clinical outcome measures in the whole schizophrenia sample (N=881) and, subsequently, in extreme groups of odor interpretation performance (best versus worst percentile), and compared them to healthy individuals (N=102).

## Method

### Subjects

#### Schizophrenia subjects

**Figure 1:**
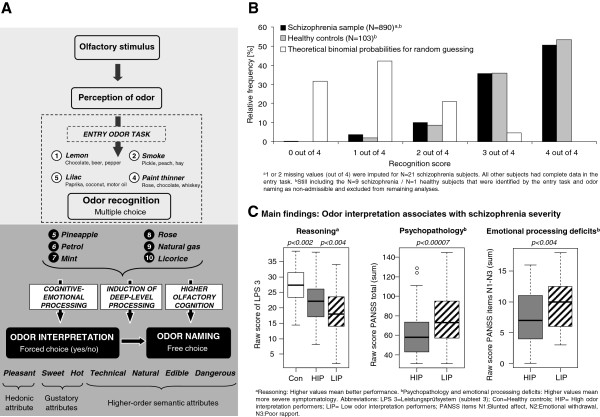
**Design of odor tasks and overview of main results. (A)** After completion of the entry odor task (passive recognition of 4 odors - multiple choice), subjects were asked to assign a set of attributes (odor interpretation) and then to name the respective odorant (odor naming), consecutively for 6 odors. **(B)** The entry odor task revealed a clear contrast between test results (schizophrenia patients, healthy controls) and theoretical binomial probabilities for guessing subjects. **(C)** The main findings of the study are summarized. Low interpretation performers (LIP) showed worse reasoning abilities, a more severe psychopathology and emotional processing deficits.

The present study (GRAS project) was approved by ethics committees of the Georg-August-University Göttingen (master committee) and collaborating centers. Detailed phenotyping of the GRAS sample [26] contained odor naming and odor interpretation tasks (Figure 1A), administered to 999 schizophrenia patients after written informed consent. Present analyses excluded all non-native German speakers (N=89), all patients with known anosmia (N=14, neurological conditions e.g. head injury, or cold) and 6 patients with missing data. Nine patients were excluded as non-admissible based on performance in entry and odor naming tasks (Figure 1B). Data analyses were based on the remaining 881 patients.

#### Healthy controls

As comparison group, 103 healthy subjects matched for age, gender and smoking status (smoker: yes/no) were recruited by public announcements and gave written informed consent. They were free of any physical, neurological and psychiatric disorder, and had no relatives with a history of neuropsychiatric diseases. One individual was excluded due to his performance in entry and odor naming tasks. Data analyses were based on 102 controls.

### Measures

#### Olfaction

##### Odorants

The University of Pennsylvania Smell Identification Test [27] (UPSIT) is the olfactory function test most widely applied clinically and scientifically. It has been used as validation criterion for newly developed olfactory tests [28]. A total of 10 different scents (scratch and sniff format) were selected from this battery (for detailed description of the administration procedure see [11]). Odorants were chosen by an experienced psychiatrist based on following criteria: (I) For the entry odor task, odors had to be easily identifiable (low item difficulty) to minimize contribution of higher cognition to task performance. For the naming task, odorants were selected to cover a broad range of difficulties to differentiate between subjects along the whole spectrum of cognitive abilities. For odor interpretation, items of low difficulty were chosen due to the expected profound interpretation deficit in schizophrenia. (II) The odors had to be of high ecological validity to German subjects. (III) To balance avoidance of redundancy (due to time limitations) and reliability, odors were selected to represent diverse associated contexts (e.g. rose and petrol) and attribute overlap (both pineapple and licorice are pleasant *and* sweet). Although this study was not designed to introduce a new *ready-to-be-used* olfaction test, preliminary item characteristics and psychometric properties are provided in Table 1.

**Table 1:** Psychometric properties of odor interpretation and odor naming tasks in the schizophrenia sample (N=881)

	**Odor naming**^**a**^	**Odor interpretation (odors)**^**b**^		**Odor interpretation (attributes)**^**b**^
***Item difficulty (weighted % correct naming ± 95%CI)***				
Pineapple	21.71 ± 0.01	73.0 ± 1.7	Pleasant	81.7 ± 1.2
Petrol	38.67 ± 0.02	78.2 ± 1.3	Sweet	72.6 ± 1.1
Mint	76.35 ± 0.03	78.0 ± 1.2	Hot	69.5 ± 1.4
Rose	39.99 ± 0.02	80.2 ± 1.3	Technical	81.3 ± 1.1
Natural gas	16.61 ± 0.02	70.9 ± 1.6	Natural	75.1 ± 1.3
Licorice	54.48 ± 0.03	82.3 ± 1.3	Edible	80.4 ± 1.2
			Dangerous	79.5 ± 1.1
***Item discrimination, part-whole corrected [95%CI]***				
Pineapple	0.24 [0.18, 0.30]	0.21 [0.15,0.27]	Pleasant	0.46 [0.40,0.51]
Petrol	0.25 [0.19, 0.31]	0.25 [0.19,0.31]	Sweet	0.33 [0.27,0.39]
Mint	0.30 [0.24, 0.36]	0.27 [0.20,0.33]	Hot	0.18 [0.11, 0.24]
Rose	0.28 [0.21, 0.34]	0.18 [0.11,0.24]	Technical	0.58 [0.53, 0.62]
Natural gas	0.13 [0.07, 0.20]	0.16 [0.09,0.22]	Natural	0.45 [0.40,0.50]
Licorice	0.27 [0.20, 0.33]	0.20 [0.14,0.27]	Edible	0.52 [0.47,0.56]
			Dangerous	0.52 [0.47,0.56]
***Item discrimination, not part-whole corrected [95%CI]***				
Pineapple	0.41 [0.35, 0.46]	0.57 [0.52,0.61]	Pleasant	0.63 [0.59,0.67]
Petrol	0.50 [0.45, 0.54]	0.53 [0.48,0.58]	Sweet	0.52 [0.47,0.56]
Mint	0.60 [0.55, 0.64]	0.52 [0.47,0.57]	Hot	0.44 [0.38,0.49]
Rose	0.56 [0.51, 0.61]	0.45 [0.40,0.50]	Technical	0.72 [0.69,0.75]
Natural gas	0.33 [0.26, 0.39]	0.50 [0.45, 0.55]	Natural	0.64 [0.60,0.68]
Licorice	0.65 [0.62, 0.69]	0.48 [0.43,0.53]	Edible	0.68 [0.64,0.71]
			Dangerous	0.66 [0.62,0.70]
***Internal consistency (standardized Cronbach’s α, equals average split-half reliability)***				
	0.50 [0.47, 0.54]	0.44 [0.40,0.48]		0.72 [0.71,0.74]

*Abbreviations:* CI, confidence interval. ^a^Psychometric measures were calculated based on Spearman correlation (ordinal data). ^b^Psychometric measures were calculated based on Pearson correlation (metric data).

##### Entry odor task

**Figure 2:**
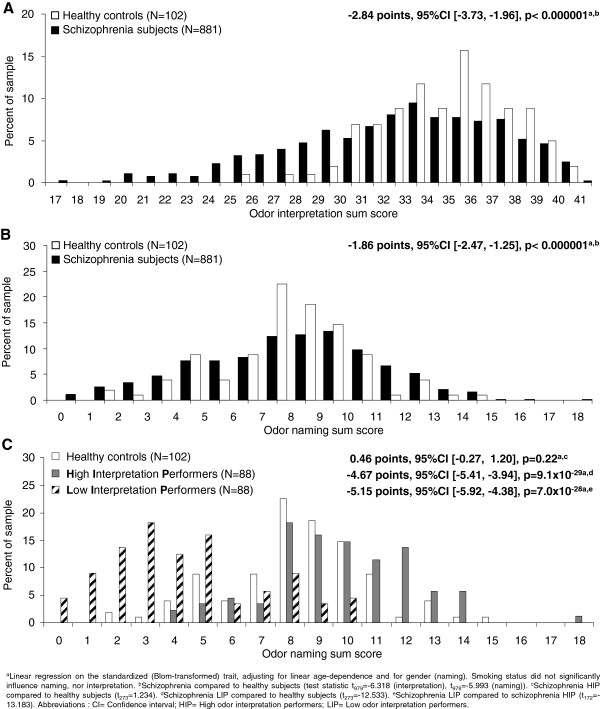
**Odor naming and interpretation sum scores in patients and healthy controls and extreme group comparison based on odor interpretation performance. (A)** Schizophrenia subjects experience severe problems in the correct interpretation of odors compared to matched healthy controls (p<0.000001). **(B)** Schizophrenia subjects are significantly impaired in the odor naming task compared to matched healthy controls (p<0.000001). **(C)** Extremely poor schizophrenia interpretation performers (LIP) display worse odor naming results compared to schizophrenia high interpretation performers (HIP) and to healthy control subjects.

To be able to interpret odor naming and interpretation results as central processing deficits, an entry odor task (Figure 1A; maximum score 4) was introduced. A total of 4 consecutively presented odors are chosen from 4 alternative descriptors each (multiple choice, brief passive odor identification [11]) by pointing to or naming the correct term on the scoring sheet. The entry task should identify potentially anosmic subjects unaware of their condition or failing to report it prior to performing odor naming and interpretation tasks. The 4 odorants *lemon*, *smoke, lilac* and *paint thinner* were chosen to be easily identifiable (Table 1) and representative of all attributes contained in the odor interpretation task. *Lemon* was selected to cover the attributes *sweet* and *edible*, *lilac* is an odor judged as *pleasant* and *natural*, *smoke* is *hot* and *dangerous* and *paint thinner* is often associated with a *technical* context. Figure 1B shows that theoretical binomial probabilities for random guessing (in case of anosmia or pronounced cognitive impairment, assuming equal multiple-choice success probabilities of 0.25 for all 4 odors) markedly differed from relative recognition score frequencies. The vast majority of patients and controls recognized 3–4 odors correctly whereas 95% of guessing subjects would recognize <3 odors correctly in the entry task. Simultaneously, guessing would result in an odor naming score of 0 (free choice test) which is extremely unlikely (Figure 2B). Consequently, subjects were excluded from analysis when they had less than 3 out of 4 correctly recognized odors in the entry task and an odor naming score of 0 (9 schizophrenia subjects, 1 healthy control subject) because of potentially experiencing difficulties at the peripheral processing level. Clearly, this approach risks excluding cognitively extremely impaired individuals. Indeed, excluded individuals cognitively underperformed the 881 schizophrenia subjects (881 subjects: -0.03 ± 0.78; 9 excluded subjects: -0.92 ± 0.54; mean ± SD of higher cognition composite; see below).

##### Odor naming and odor interpretation

The odor naming task (maximum score 18) measures the correct naming of the 6 odors *pineapple, petrol, mint, rose, natural gas* and *licorice* (free choice; Figure 1A). Three points were scored for the correct name, 1 point if the subject provided an item belonging to the same semantic category (e.g. *pineapple*: 3 points for ‘pineapple’, 1 point for ‘fruit’). Before naming an odor, probands had to decide whether it matched the 7 attributes *pleasant* (hedonic judgement)*, sweet* &*hot* (gustatory judgement)*, technical, natural, edible* and *dangerous* (semantic judgement) (forced choice: ‘yes’ or ‘no’ answer; odor interpretation; same set of odors as for naming) (Figure 1A). One point was scored for each attribute assignment consistent with the predefined profile (maximum score 42=6x7). Although task reliability improves with number of items [29], we had to restrict it to 6 items for time-economic reasons (olfactory tasks being only a fraction of the GRAS examination procedure [26]).

#### Disease-relevant possible confounders

As unspecific but disease-relevant (overall severity) possible confounders, duration of disease, number of hospitalizations and medication status (chlorpromazine equivalents) were selected.

#### Functional outcome, disease severity, psychopathology, cognition and emotional processing

To address schizophrenia relevant target domains, age-at-prodrome, general assessment of functioning (GAF) scale, PANSS (positive and negative syndrome scale) subscales [30], a higher cognition composite (comprising neuropsychological measures listed in Table 2), and the alertness subtest (mean of tonic and phasic alertness) of TAP (Testbatterie für Aufmerksamkeitsprüfung [31], English translation: Test battery for attention) were included. MacQuarrie dotting and tapping tests [32] were integrated into a fine motor function composite. The Mehrfachwahl-Wortschatz-Test [33] (MWTB, English translation: Multiple Choice Vocabulary Test of Intelligence) covered premorbid intelligence. Emotional processing was operationalized as sum score of ‘blunted affect’, ‘emotional withdrawal’ and ‘poor rapport’ of PANSS, as previous studies have shown that amygdala responses to facial emotional expression correlate with the negative subscale of PANSS [34]. Higher scores represent worse outcome in PANSS but better performance in GAF and neuropsychological tests. Healthy controls completed odor naming and interpretation tasks and 4 neuropsychological tests (LPS 3 [35]; MWTB [33]; subtests dotting and tapping subtests of the MacQuarrie test for mechanical ability [32]). All tests are cited in [26].

**Table 2:** Presentation of high (HIP) and low (LIP) interpretation performer profiles with respect to higher cognition composite components and emotional processing items

			** Descriptive**		** LIP compared to HIP**	
		**SZ**	**LIP**	**HIP**	**Effect**	**p**
					**[95%CI]**	**(Statistic)**
**Higher cognition composite components**						
Reasoning^c^	mean ± SD	20.8 ± 6.6	18.2 ± 6.7	21.5 ± 6.5	−2.61	**3.6x10**^**-3**^
	range	(2–38)	(2–34)	(8–38)	[−4.35, -0.86]	(t_164_=−2.951^a^)
Executive functioning^c^	median	−56.5	−79.0	−47.0	−24.77	0.082
	interquartile	[−103.0, -36.0]	[−139.0, -47.0]	[−122.0, -34.0]	[−52.69, 3.15]	(t_155_=−1.753^a^)
	range	(−868– 40)	(−680– -9)	(−563– -10)		
Working memory^c^	mean ± SD	13.1 ± 3.9	11.7 ± 4.4	13.6 ± 3.9	−1.25	*0.049*
	range	(1–24)	(4–22)	(4–24)	[−2.50, -0.00]	(t_158_=−1.981^a^)
Processing speed^c^	mean ± SD	38.4 ± 13.2	32.5 ± 13.7	39.3 ± 13.5	−4.52	*0.011*
	range	(4–88)	(5–68)	(6–71)	[−7.99, -1.04]	(t_167_=−2.566^a^)
Verbal memory^c^	mean ± SD	42.0 ± 12.6	37.3 ± 12.5	43.0 ± 14.0	−3.38	0.083
	range	(6–72)	(11–62)	(6–70)	[−7.22, 0.45]	(t_161_=−1.742^a^)
Divided attention^c^	median	−725	−764	−714	4.14	0.84
	interquartile	[−806, -659]	[−833, -651]	[−797, -647]	[−35.50, 43.78]	(t_158_= 0.206^a^)
	range	(−1663– -355)	(−1167– -448)	(−1160– -416)		
**Emotional processing items**						
Blunted affect	median	3	4	3		**7.5x10**^**-3**^
(PANSS N1)	interquartile	[2,4]	[3,5]	[2,4]	-	(W=2947^b^)
	range	(1–7)	(1–7)	(1–6)		
Emotional withdrawal	median	3	3	3		*0.029*
(PANSS N2)	interquartile	[1,4]	[1,4]	[1,4]	-	(W=3119^b^)
	range	(1–7)	(1–7)	(1–6)		
Poor rapport	median	2	3	2		**3.1 x10**^**-3**^
(PANSS N3)	interquartile	[1,3]	[1,4]	[1,3]	-	(W=2875^b^)
	range	(1–7)	(1–6)	(1–5)		

Multiple testing adjusted significances are set in boldface (p≤0.0083 for components of higher cognition composite, p≤0.0167 for emotional processing items). All other p-values≤0.05 in italic. ^a^t-Statistic t_df_ with df degrees of freedom for estimation of difference in means (employing a linear model on Blom-transformed trait). ^b^Wilcoxon rank sum test. ^c^Adjusted for age and PANSS negative. Reasoning ability was measured by LPS3, executive functioning is represented as difference of execution times between TMTA and TMTB, working memory was assessed by BZT, processing speed by ZST, verbal memory by VLMT and divided attention by negative execution time (−d3mdg) of TAP. In this table, and when building the cognition composite, all measures were presented such that larger values represent better cognitive performance. *Abbreviations:* SZ= Descriptive statistics for schizophrenia sample (N=881); HIP= High interpretation performers (N=88); LIP= Low interpretation performers (N=88); LPS3= Subtest 3 of the Leistungsprüfsystem, a German test covering Thurstone’s primary mental abilities; TMTA/B= Trail Making Test A and B; BZT=Letter Number Sequencing; ZST= Digit Symbol Coding; VLMT= Verbal Learning and Memory Test; TAP=Testbatterie für Aufmerksamkeitsprüfung, English translation: Test battery for attention. All neuropsychological measures are cited in Ribbe et al. 2010.

### Statistical analyses

To test for associations of odor naming and interpretation performance with disease-relevant symptom domains, multiple linear regression was applied within the schizophrenia sample (Table 3). The model simultaneously tested for linear dependence of the target (odor naming or odor interpretation performance) on a set of multiple predictor variables (cognitive performance, symptom severity and control variables) while adjusting for gender (odor naming) and for linear age-dependence (odor naming and odor interpretation). Displayed regression coefficients are mutually adjusted for the respective other predictors. All variables were a priori standardized such that regression coefficients quantify relative association strengths interpretable in analogy to Cohen’s d, displaying mutually adjusted *relative* association strengths interpretable in analogy to Cohen’s d). Sum scores for higher cognition and fine motor performance are the mean of standardized neuropsychological measures in these domains (larger values represent better performance). Rank-based Blom transformation [36] was applied to standardize all measures by transforming them into standard normally distributed surrogates prior to sum score computation. This maintained the order of the data, but removed skewness from variable distributions. Group comparisons between healthy and schizophrenia subjects (Figure 2A-B), and schizophrenia subgroups (Figure 1C, Figure 2C and Tables 2, 4 and 5) were tested by linear regression (for standardized normally distributed quantitative targets, adjusting for covariates where indicated), Fisher’s exact test (for binary targets gender, smoking and genetic risk status), or non-parametric Wilcoxon rank sum test (for quantitative targets which could not be standardized to normal distribution). For linear model analyses, the target variable is denoted in the table row, respectively. The tested predictor was class membership, adjusting for linear dependence on age, PANSS negative and for gender where indicated. Standardized regression coefficients (with 95% confidence limits) were converted to the original variable scale for ease of interpretation by multiplication with the raw data standard deviation of the target variable within the respective data. Multiple-testing was accounted for by Bonferroni correction and closed testing principle (Table 4). All p-values are two-sided.

**Table 3:** Odor naming is associated with cognition and odor interpretation with severity of positive symptoms and alertness in schizophrenia subjects (N=881)

	**Odor naming**^**a**^	**Odor interpretation**^**a**^
Higher cognition composite^b^	**0.165**	0.057
	**(t**_**813**_**= 2.347, p=0.019)**	(t_814_= 0.789, p=0.430)
Alertness (TAP)	**0.144**	**0.196**
	**(t**_**813**_**= 3.401, p=0.001)**	**(t**_**814**_**= 4.635, p=0.000)**
PANSS negative	**−0.112**	−0.045
	**(t**_**813**_**=−2.717, p=0.007)**	(t_814_=−1.073, p=0.283)
PANSS positive	−0.055	**−0.107**
	(t_813_=−1.407, p=0.160)	**(t**_**814**_**=−2.701, p=0.007)**
Fine motor function composite^c,d^	−0.010	0.017
	(t_813_=−0.210, p=0.834)	(t_814_= 0.336, p=0.737)
Premorbid intelligence (MWTB)^d^	0.071	0.059
	(t_813_= 1.761, p=0.079)	(t_814_= 1.420, p=0.156)

^a^Relative association strength of domains with odor naming and odor interpretation (t-statistic t_df_ with df degrees of freedom, p value). Multiple linear regression model containing linear terms for all six predictor variables (table rows) and adjusting for age (odor naming and odor interpretation) and gender (odor naming), significances (Bonferroni p≤0.025) in boldface. ^b^The higher cognition composite includes the following cognitive domains: Reasoning ability (subtest 3 of the *Leistungsprüfsystem*, a German test covering Thurstone’s primary mental abilities), executive functioning (difference of execution times between Trail Making Test A and B), working memory (Letter Number Sequencing), processing speed (Digit Symbol Coding), verbal memory (Verbal Learning and Memory Test) and divided attention (test battery for attention). ^c^The fine motor function composite consists of dotting and tapping subtests of the MacQuarrie test for mechanical ability. ^d^Control variables.

**Table 4:** Severely impaired interpretation performers (LIP) show compromised cognition compared to healthy controls (Con) and non impaired interpretation performers (HIP)

**Trait**	**Descriptive**			**Group contrast**	**Effect**	**[95%CI]**	**Statistic**	**p**
**Basic characteristics**								
Age (years)	SZ	39.5 ± 13	(17–78)					
mean ± SD (range)	Con	38.8 ± 14	(18–71)	HIP / Con	2.75	[−0.81, 6.31]	t_275_= 1.522^a^	0.13
	HIP	41.0 ± 11	(22–64)	LIP / Con	1.25	[−2.31, 4.81]	t_275_= 0.693^a^	0.49
	LIP	39.7 ± 13	(18–71)	LIP / HIP	−1.40	[−4.58, 1.77]	t_174_=−0.873^a^	0.38
Gender (% female)	SZ	33						
	Con	32		HIP / Con	OR 1.25	[0.66, 2.38]	Fisher^b^	0.54
	HIP	38		LIP / Con	OR 0.83	[0.42, 1.62]	Fisher^b^	0.64
	LIP	28		LIP / HIP	OR 0.66	[0.33, 1.31]	Fisher^b^	0.26
					for females			
Smoker status (% yes)	SZ	48						
	Con	63		HIP / Con	*OR 0.45*	[0.21, 0.94]	Fisher^b^	*0.025*
	HIP	43		LIP / Con	OR 0.57	[0.26, 1.23]	Fisher^b^	0.15
	LIP	49		LIP / HIP	OR 1.26	[0.52, 3.05]	Fisher^b^	0.68
					for smokers			
**Disease related higher and basal cognition and premorbid intelligence**								
Reasoning^c^	SZ	20.8 ± 6.6	(2–38)					
mean ± SD (range)	Con	27.2 ± 5.2	(14–38)	HIP / Con	−3.00	[−4.86, -1.14]	t_265_=−3.180^a^	**1.6x10**^**-3**^
	HIP	21.5 ± 6.5	(8–38)	LIP / Con	−5.49	[−7.63, -3.35]	t_265_=−5.058^a^	**7.9 x10**^**-7**^
	LIP	18.2 ± 6.7	(2–34)	LIP / HIP	−2.45	[−4.07, -0.82]	t_164_=−2.971^a^	**3.4 x10**^**-3**^
Fine motor function	SZ	−0.12 ± 0.91	(−3.22–3.22)					
composite^c^	Con	0.97 ± 0.68	(−1.09–2.83)	HIP / Con	−0.53	[−0.75, -0.31]	t_268_=−4.674^a^	**4.7 x10**^**-6**^
mean ± SD (range)	HIP	−0.002 ± 0.87	(−2.40–1.83)	LIP / Con	−0.78	[−1.03, -0.52]	t_268_=−5.951^a^	**8.3 x10**^**-9**^
	LIP	−0.45 ± 0.93	(−2.89–1.90)	LIP / HIP	−0.23	[−0.41, -0.05]	t_167_=−2.471^a^	*0.014*
Premorbid	SZ	26.2 ± 6.1	(4–37)					
intelligence^d^	Con	30.9 ± 4.0	(18–37)	HIP / Con	−0.22	[−2.06, 1.62]	t_267_=−0.236^a^	0.81
mean ± SD (range)	HIP	27.8 ± 5.0	(13–37)	LIP / Con	−2.60	[−4.74, -0.46]	t_267_=−2.393^a^	*0.017*
	LIP	23.8 ± 7.0	(5–35)	LIP / HIP	−2.51	[−4.19, -0.83]	t_166_=−2.955^a^	**3.6 x10**^**-3**^

Multiple testing adjusted significances (applying Bonferroni (p≤ 0.007) and the closed testing principle) are set in boldface. All other p-values 0.05 are set in italic; ^a^t-Statistic t_df_ with df degrees of freedom for estimation of difference in means (employing a linear model on Blom-transformed trait); ^b^Fisher’s exact test for count data; ^c^Adjusted for age at exam and PANSS negative (value 7 for healthy control). Reasoning ability was measured by LPS3 (subtest 3 of the *Leistungsprüfsystem*, a German test covering Thurstone’s primary mental abilities). Fine motor function composite is the mean from standardized (by Blom-transformation) MacQuarrie dotting and tapping; ^d^Adjusted for age (age of onset of psychosis for schizophrenia HIP and LIP, age at exam for healthy control) and PANSS negative (value 7 for healthy control). Premorbid intelligence was assessed by MWTB (Mehrfachwahl-Wortschatz-Intelligenztest, English translation: multiple choice vocabulary test of intelligence). Abbreviations: Con= Healthy control (N=102); SZ= Descriptive statistics for whole schizophrenia sample (N=881); HIP= High interpretation performers (N=88); LIP= Low interpretation performers (N=88), OR=Odds Ratio.

**Table 5:** Severely impaired interpretation performers (LIP) show a more severe psychopathology, compromised cognition and emotional processing compared to high interpretation performers (HIP)

			**Descriptive**		** LIP compared to HIP**	
		**SZ (N=881)**	**LIP (N=88)**	**HIP (N=88)**	**Effect [95%CI]**	**P (Statistic)**
**Disease relevant confounders**						
Years of education	median	12.0	11.0	13.0	−1.55	**5.4 x10**^**-4**^
(years)	interquartile	[10.0, 13.5]	[9.0,13.0]	[10.0,15.0]	[−2.42, -0.68]	(t_174_=−3.525 ^a^)
	range	(8.0-27.0)	(8.0-20.5)	(8.0-24.0)		
Duration of disease	median	11.0	13.7	11.1		0.28
(years)	interquartile	[4.7, 20.1]	[6.4, 21.9]	[5.3, 20.9]	-	(W=3466^b^)
	range	(0.008,58.4)	(0.1, 47.4)	(0.6, 45.3)		
Number of	median	3.0	3.0	4.0		0.10
hospitalizations	interquartile	[2.0, 6.0]	[2.0, 5.0]	[3.0, 6.0]	-	(W=2532^b^)
	range	(0, 55)	(1, 44)	(1, 50)		
Chlorpromazine	median	500	600	450		0.068
equivalents	interquartile	[250, 900]	[300, 1072]	[228, 817]	-	(W=3216^b^)
	range	(0, 7375)	(0, 3238)	(0, 3064)		
**Functional outcome, disease severity and psychopathology**						
General assessment of	mean ± SD	45.6 ± 17.2	38.0 ± 17.1	49.8 ± 17.0	−11.93	**6.5x10**^**-6**^
functioning (GAF)	range	(5, 90)	(10, 85)	(20, 90)	[−16.99, -6.87]	(t_171_=−4.654^a^)
Age-at-prodrome (years)	median	20	19	23	−2.70	0.054
	interquartile	[17,26]	[16,24]	[17,30]	[−5.44, 0.05]	(t_153_=−1.942^a^)
	range	(2, 66)	(12, 66)	(2, 47)		
PANSS negative	median	17.0	22.0	15.0	4.54	**1.7 x10**^**-4**^
	interquartile	[12.0, 23.0]	[14.0, 27.0]	[11.0, 21.8]	[2.21, 6.88]	(t_171_= 3.840^a^)
	range	(7.0, 44.0)	(7.0, 40.0)	(7.0, 38.0)		
PANSS positive	median	12.0	15.0	11.0	3.96	**1.6 x10**^**-4**^
	interquartile	[9.0, 17.0]	[10.0, 22.0]	[8.0, 15.8]	[1.94, 5.98]	(t_171_= 3.869^a^)
	range	(7.0, 38.0)	(7.0, 37.0)	(7.0, 38.0)		
**Disease-related cognition**						
Higher cognition	mean ± SD	−0.03 ± 0.78	−0.41 ± 0.84	0.07 ± 0.83	−0.24	**4.3 x10**^**-3**^
composite^c^	range	(−2.64, 1.93)	(−2.64, 1.40)	(−1.92, 1.77)	[−0.40, -0.07]	(t_169_=−2.896^a^)
Alertness (TAP)^c^	median	−282	−331	−270	−80.75	**1.4 x10**^**-4**^
(msec)	interquartile	[−350, -245]	[−479, -270]	[−311, -237]	[−121.61, -39.89]	(t_164_=−3.902^a^)
	range	(−1288, -163)	(−1219, -206)	(−878, -192)		
**Emotional processing items**						
PANSS negative	median	8.0	10.0	7.0		**3.3 x10**^**-3**^
N1+N2+N3^d^	interquartile	[5.0, 11.0]	[6.0, 12.3]	[4.0, 11.0]	-	(W=2883^b^)
	range	(0.0, 21.0)	(3.0, 18.0)	(0.0, 16.0)		

Multiple testing adjusted significances (Bonferroni: p≤0.0045) are set in boldfaces, all other p-values≤0.05 in italic. ^a^t-Statistic t_df_ with df degrees of freedom for estimation of difference in means (employing a linear model on Blom-transformed trait; small differences in degrees of freedom are due to low percentages of missing trait values). ^b^Wilcoxon rank sum test. ^c^Adjusted for age and PANSS negative. ^d^N1: Blunted affect, N2: Emotional withdrawal, N3: Poor rapport. *Abbreviations:* SZ= Descriptive statistics for whole schizophrenia sample (N=881); HIP= High interpretation performers (N=88); LIP= Low interpretation performers (N=88), OR=Odds Ratio.

## Results

### Influence of age, smoking and gender on odor naming and interpretation

Increasing age is associated with a decline in odor naming (average −0.05 points/year, p=2.2x10^-11^, test statistic t_978_=−6.770) and interpretation performance (average −0.06 points/year, p=1.6×10^-8^, t_979_=−5.697) likewise in schizophrenia and healthy individuals (no significant difference). Smoking status had no influence on both central olfactory measures. Women proved to be slightly superior to men in odor naming (average 0.42 points, p=0.043, t_978_=2.022) but not interpretation.

### Odor naming and interpretation are impaired in schizophrenia patients

The main study results are summarized in Figure 1C. Inferior performance of schizophrenia patients (N=881) compared to 102 matched healthy controls became evident for odor interpretation and naming tasks (Figure 2A,B; interpretation: p=4.0×10^-10^; naming: p=2.9×10^-9^).

### Odor naming and interpretation are differentially associated with psychopathology and cognition in schizophrenia patients

Odor naming and interpretation correlate substantially (Spearman correlation r=0.51, 95%CI [0.46, 0.56], p<2.2×10^-16^). To investigate relative association strengths of odor naming and interpretation with 3 schizophrenia relevant symptom domains (cognition, negative and positive symptoms) and 2 control variables (fine motor function and premorbid intelligence) (Table 3), multiple linear regression was applied in the schizophrenia sample (N=881). Regression coefficients express *relative* association strength (relative to the standard deviation of the target trait). Odor naming and interpretation scores were strongly dependent on alertness. Worse naming results were additionally linked to impaired higher cognition and more severe negative symptoms (PANSS). Deficits in odor interpretation were associated with higher positive symptom severity (PANSS). No significant link was detected between odor naming or interpretation and fine motor function or premorbid intelligence.

### Extreme group comparisons based on odor interpretation performance reveal strong differences in several lead symptom domains

The frequency distributions of interpretation and naming scores (Figure 2A,B) showed increased probabilities of low scores for schizophrenia patients, especially for odor interpretation. This suggests that olfactory processing deficits may be particularly pronounced in a subgroup of schizophrenia patients. To achieve a maximum phenotypic contrast, schizophrenia high interpretation performers (HIP, scoring >90^th^ percentile) were contrasted with low interpretation performers (LIP, scoring <10^th^ percentile; N=88 each) and compared to healthy controls (Con, N=102) with respect to basic sample characteristics, disease related higher and basal cognition and premorbid intelligence. The 3 groups did not differ (pairwise comparisons) with regard to age, gender and smoking status. Expectedly, a highly significant naming deficit became obvious for LIP in comparison to HIP (p=7.0×10^-28^) and healthy controls (p=9.1×10^-29^) (Figure 2C). In terms of reasoning capabilities, healthy controls obtained better results than schizophrenia HIP (p=1.6×10^-3^) with the latter performing superior to LIP (Table 4, p=3.4×10^-3^) (Figure 1C). For fine motor function, both schizophrenia groups performed comparably and markedly worse than healthy controls (HIP/LIP: p=0.015; HIP/Con: p=4.4×10^-6^; LIP/Con: p=8.1×10^-9^). Also for premorbid intelligence, a difference was observed between disease groups (HIP/Con: p=0.81; LIP/Con: p=0.017; LIP/HIP: p=3.6×10^-3^) (Table 4).

More detailed assessment of group contrast between schizophrenia LIP compared to HIP in disease-related domains, revealed higher severity of negative symptoms (p=1.7×10^-4^) and positive symptoms (p=1.6×10^-4^) for LIP (Figure 1C). Additionally, strongly impaired cognitive and emotional processing became evident (higher cognition composite: p=3.7×10^-3^; alertness: p=1.4×10^-4^; emotional processing: p=3.3×10^-3^), resulting in a worse functional outcome (p=6.5×10^-6^; average difference 12 GAF units) for LIP (Table 5). Extreme group comparison for individual neuropsychological measures (Table 2) revealed strongest differences for reasoning (p=3.6×10^-3^) followed by processing speed (p=0.011) and working memory (p=0.049). Results for executive functioning, verbal memory and divided attention failed to be significant. No significant differences were observed with regard to possible confounders indicating overall disease severity (duration of disease, number of hospitalizations and chlorpromazine equivalents). Also, age-at-prodrome (median, range for LIP: 19, 12–66, for HIP: 23, 2–47, p=0.054) and age-of-onset (LIP: 23, 15–67, HIP: 27, 8–55, p=0.0079) were only close to or nominally significant, i.e. above the Bonferroni corrected significance threshold (p>0.005). Together, the results of the extreme group approach indicate that deficits in odor interpretation are accompanied by more severe psychopathology, worse cognitive and emotional processing and inferior functional outcome.

## Discussion

The present study, building on and extending previous work, demonstrates in a large sample of subjects that the ability to name and qualitatively interpret odors is markedly impaired in schizophrenia. Importantly, the study adds new aspects to the available literature by showing distinct associations between central olfactory measures and schizophrenia-relevant clinical variables. Extreme group comparisons providing maximum phenotypic contrast deliver further evidence for a biological subgroup of patients, defined by a severe olfactory dysfunction. This subgroup is characterized by profound abnormalities in higher olfactory processing, together with a more severe psychopathology, remarkable cognitive deficits across various neuropsychological measures and pronounced affective flattening, resulting in an overall inferior functional outcome as compared to all other schizophrenia patients.

### Odor naming ability relies on higher cognitive processing

Odor naming but not interpretation was found to depend on the integrity of higher cognitive processing when analyzed in all 881 patients. In contrast to Saoud and coworkers, who investigated only a small number of individuals [16], we found odor naming, as almost all neuropsychological tests [26], to correlate with PANSS negative scores. Due to the low number of odors used for the naming task in the present work, distributions of odor naming sum scores were not considered suitable for the definition of extreme groups. Instead, the definition was based on interpretation performance. Future studies will have to aim at doubling the number of odors to be able to thoroughly explore construct validity of the odor naming task.

### Positive symptoms interfere with odor interpretation ability

By applying multiple linear regression analyses to investigate relative association strengths of olfactory target measures with several symptom domains, odor interpretation, but not naming, was found to be influenced by the severity of positive symptoms. In fact, while the relationship between basic olfactory processing (e.g. passive odor identification) and severe negative symptoms is quite well established [37-41], this is the first study to provide evidence for psychotic symptoms to interfere with performance in an olfactory function test. This relationship is supported by imaging studies showing that functional abnormalities in temporo-limbic regions are related to altered familiarity and hedonicity judgements in schizophrenia [42]. Disconnectivity in these brain regions also contributes to the emergence of hallucinations in schizophrenia [43,44]. Based on these findings future studies should address whether the relationship of odor interpretation and positive symptom severity is mediated by a dysfunctional integration of sensory input into cognitive-emotional processing. The potential for odor interpretation tasks to indirectly assess the predisposition to develop positive symptoms should also be further evaluated.

### Alertness modulates performance in central olfactory measures

The large amount of variance shared by odor naming and interpretation can be partially explained by the strong association of both olfactory tasks with the level of alertness. The latter represents the intensity aspect of attention and comprises both a state of general wakefulness (intrinsic alertness) and the ability to increase the readiness to react in response to a cue over a short period of time (phasic alertness) [45]. Importantly, attention is a requirement for assigning the continuous flow of air through the nose to either respiration or olfactory exploration [46]. Non-attended, the olfactory content of inhalations is ignored and not processed further. Effects of attentional modulation on activity in human primary [46] and secondary olfactory cortices [47] and amygdala [48] support this notion. Consequently, the extent to which an individual attends to a presented odor and is able to maintain the level of alertness during the completion of a task, likely influences the amount of resources provided for higher-order mental operations following the perception of an olfactory stimulus. Additionally, it is well established that olfactory (spicy, fruity) and gustatory (salty, sweet) judgements lead to the activation of associated semantic networks. This results in a facilitation of olfactory naming – at least in healthy individuals [25]. However, as the here applied odor interpretation task was the same for all odors, differential depth-of-processing effects, potentially confounding odor naming results, can be excluded.

### Odor interpretation is associated with discrete neuropsychological functions

Comparisons of low and high odor interpretation performers (HIP / LIP) with regard to single neuropsychological measures revealed largest group differences for reasoning abilities. Processing speed and working memory also differed significantly dependent on odor interpretation performance whereas executive functioning (set shifting), verbal memory and divided attention did not. Together, these findings should stimulate further research into primary cognitive functions involved in the odor interpretation task.

Of note is the fact that premorbid intelligence, age-at-prodrome and age-of-onset of schizophrenia showed at least a strong tendency of a difference between LIP and HIP, indicating a potentially more pronounced neurodevelopmental aspect of the disease in LIP. This difference alone, however, is unlikely to explain the marked contrast in psychopathology and cognition between extreme groups since post hoc linear regression models remained significant upon inclusion of neurodevelopmental variables as covariates (data not shown).

## Conclusions

Although further work is needed to improve psychometric properties of the measures by e.g. increasing the number of applied odors, we deliver first evidence for both tasks to accentuate slightly dissimilar aspects of higher brain functioning. Future studies are needed to delineate whether prevailing impairment in either odor naming or interpretation indicates a preferential lack of integrity in either sensory-cognitive or sensory-emotional brain networks. If developed into psychometrically sound tests, odor naming and interpretation tasks could assist the delineation of biologically defined disease subphenotypes, characterized by preponderance of either cognitive decline/negative symptoms, or of pronounced positive symptoms.

## Competing interest

The authors declare that they have no competing interests.

## Authors’ contributions

AK, MB and HE developed the olfactory tasks. Phenotype data were collected and entered into the database by CH, MB and AK. DM and AK performed all statistical analyses and designed manuscript and figures. HE guided the project and data analysis, together with HB. AK, together with HE, wrote the paper. All authors discussed the results, read and commented on the manuscript, and have seen and approved the final version.

## Pre-publication history

The pre-publication history for this paper can be accessed here:

http://www.biomedcentral.com/1471-244X/13/218/prepub
